# Sequences encoding C2H2 zinc fingers inhibit polyadenylation and mRNA export in human cells

**DOI:** 10.1038/s41598-018-35138-4

**Published:** 2018-11-19

**Authors:** Joseph Russo, Aimee L. Jalkanen, Adam M. Heck, Caleb M. Schmidt, Jeffrey Wilusz, Carol J. Wilusz

**Affiliations:** 1 Department of Microbiology, Immunology & Pathology, Colorado State University, Fort Collins, Colorado USA; 2 Program in Cell & Molecular Biology, Colorado State University, Fort Collins, Colorado USA

## Abstract

The large C2H2-Zinc Finger (C2H2-ZNF) gene family has rapidly expanded in primates through gene duplication. There is consequently considerable sequence homology between family members at both the nucleotide and amino acid level, allowing for coordinated regulation and shared functions. Here we show that multiple C2H2-ZNF mRNAs experience differential polyadenylation resulting in populations with short and long poly(A) tails. Furthermore, a significant proportion of C2H2-ZNF mRNAs are retained in the nucleus. Intriguingly, both short poly(A) tails and nuclear retention can be specified by the repeated elements that encode zinc finger motifs. These Zinc finger Coding Regions (ZCRs) appear to restrict polyadenylation of nascent RNAs and at the same time impede their export. However, the polyadenylation process is not necessary for nuclear retention of ZNF mRNAs. We propose that inefficient polyadenylation and export may allow C2H2-ZNF mRNAs to moonlight as non-coding RNAs or to be stored for later use.

## Introduction

Zinc-finger (ZNF)-containing proteins are among the largest families of proteins in the human genome. The Cys2His2 (C2H2)-type ZNF family has over 700 members, many of which are unique to primates and have arisen through gene duplication^1,2^. The vast majority of C2H2-ZNF proteins have no unique biological function assigned to them; however, they are widely accepted as DNA binding proteins and those with an associated KRAB domain can recruit the transcriptional repressor KAP1/TRIM28^3^. Recent studies have suggested that expansion of this family in primates occurred in part to combat the parallel expansion and evolution of transposable elements^4–7^. Notably, the human C2H2-ZNF genes have on average ~10 tandem copies of the ZNF motif^2^, which is sufficient to recognize a 30nt sequence, far longer than necessary for specificity^8^. Indeed, only ~60% of ZNF motifs were predicted to bind DNA in a recent analysis^9^. This gives rise to the possibility that some ZNF domains, or perhaps even the sequences encoding them, have other roles.

In support of the zinc finger motif having additional functions, many C2H2-ZNFs were recently identified as putative RNA-binding proteins (RBPs)^10^ and the C2H2-ZNF motif has also been implicated in protein-protein interactions^11,12^. In addition, the linker domains between individual ZNFs are targeted by the TOPK/PBK kinase during mitosis to induce dissociation of these proteins from condensing chromatin^13,14^. Thus, these highly conserved repeated protein motifs may indeed serve functions other than binding DNA.

It is also possible that the DNA or RNA sequences encoding C2H2-ZNF domains have an additional, non-coding function. Sequence elements shared between different genes can facilitate coordinated control through recruitment of regulatory factors (such as RBPs and miRNAs). Several lines of evidence suggest that C2H2-ZNF genes share regulatory elements that allow their expression to be coordinated. First, four miRNAs can target the repeated regions encoding zinc fingers found in related C2H2-ZNF mRNAs^15,16^. These miRNAs can simultaneously downregulate multiple C2H2-ZNF genes through translational repression and/or mRNA decay^15,16^. Second, C2H2-ZNF mRNAs are over-represented among transcripts with shorter than average poly(A) tails which could indicate unique processing or deadenylation mechanisms are at play^17^. Third, a large group of C2H2-ZNF transcripts are decayed more slowly in stem cells than in fully differentiated fibroblasts^18^. This could be facilitated by recognition of shared sequence elements by a stem cell or fibroblast-specific RBP or miRNA. Fourth, mRNAs encoding KRAB C2H2-ZNFs share similar RNA metabolism profiles including efficient synthesis and processing, and a tendency to be retained in the nucleus^19^. Next, the 3′ exons of C2H2-ZNF genes have unusual chromatin modification (H3me3K9) suggesting that they may auto-regulate through recruitment of the KAP1/TRIM28 transcriptional repressor^20,21^. Interestingly the level of H3me3K9 modification is strongly correlated with the number of ZNF motifs in the 3′ exon^21^. Finally, C2H2-ZNF genes are significantly over-represented among genes that have acquired *Alu* insertions^22^ which again may facilitate coordinated control either at the level of transcription or through ADAR-editing^23^.

Based on the evidence outlined above, we hypothesized that sequence elements found within the ORF or 3′UTR of C2H2-ZNF mRNAs are responsible for aspects of their unique metabolism. We focused specifically on the observations made through global analyses that many C2H2-ZNF mRNAs have significant populations with shorter than average poly(A) tails^17^ and that they have an increased propensity to be retained in the nucleus^19^. In mammalian cells, a poly(A) tail of ~250 nt is added to the 3′ end of the majority of nascent mRNAs^24^ and influences downstream metabolism. Failure to polyadenylate a nascent transcript limits its ability to form a RiboNucleoprotein Complex (RNP) that is competent for export and leads to rapid decay^25^. Once a polyadenylated transcript has been exported, the poly(A) tail, in complex with PABPC1, potentiates translation by helping to recruit initiation factors. Over time, the poly(A) tail can be shortened by various deadenylases^26^. When the tail is shortened to such an extent that PABPC1 can no longer bind, rapid decay of the mRNA body generally ensues^27^. Thus at steady state, most mRNAs exist as a population with poly(A) tails varying in length from ~20 to ~250 nt^28,29^. Exceptions to this are the histone mRNAs^30^, transcripts with poly(A) limiting elements (PLEs)^31,32^ and mRNAs bearing cytoplasmic polyadenylation elements (CPEs)^33^ which can persist with short or no poly(A) tail. Here, we show that repetitive sequence elements encoding C2H2-ZNF motifs (Zinc finger Coding Regions or ZCRs) act as poly(A) limiting elements, resulting in a sub-population of mRNAs that have short poly(A) tails. In addition, we provide evidence that C2H2-ZNF mRNAs are retained in the nucleus through mechanisms that do not require polyadenylation.

## Results

### The poly(A) tails of C2H2-ZNF mRNAs are bimorphic

Most mRNAs can be effectively captured by hybridization of the poly(A) tail to oligo(dT)^34^. In contrast, histone mRNAs and many non-coding RNAs (rRNAs, tRNAs etc) fail to bind to oligo(dT) because they lack a poly(A) tail. Yang *et al*.^17^ identified a group of mRNAs in human H9 embryonic stem cells and HeLa cells that are “bimorphic”, in that a significant portion of the population fails to bind oligo(dT). Among these are many C2H2-ZNF mRNAs.

We evaluated six C2H2-ZNF mRNAs with different properties (numbers of ZNF motifs, +/−KRAB effector domain, chromosomal locations, etc), some of which were previously identified as bimorphic^17^ and most of which share similar metabolism profiles (“biotypes”) as classified by Mukherjee *et al*.^19^ who evaluated various properties of mRNAs including nuclear/cytoplasmic distribution, synthesis, processing, translation and decay rates (Table 1).

**Table 1:** Properties of C2H2-ZNF genes analyzed in this study.

Gene Name	Gene ID	Genomic Location	Number of ZNF Motifs^a^	Poly(A) Status^b^	Biotype Cluster^c^	ORF miRNA Binding Sites^d^
ZNF2	7549	2q11.1	9	Bimorphic (HeLa)	c4	4 miR-188, 6 miR-199
ZNF12	7559	7p22.1	14	Bimorphic (HeLa/H9)	c3	20 miR-181, 10 miR-188
ZNF43	7594	19p12	22	Bimorphic (H9)	c3	19 miR-23
ZNF134	7693	19q13.43	11	Bimorphic (H9)	c3	8 miR-188
ZNF280B	140883	22q11.22	2	Unknown	c3	0
ZNF627	199692	19p13.2	12	Unknown	c3	7 miR-188, 7 miR-199

^a^The number of ZNF motifs is taken from predictions of functional C2H2-ZNFs made by NCBI Conserved Domain Search^86^, additional ZNF-related motifs may be present upon close inspection.

^b^Poly(A) status as determined by Yang *et al*.^17^.

^c^Mukherjee *et al*. classified human coding and non-coding transcripts into 7 groups based on RNA metabolism profiles. KRAB-domain ZNFs were enriched in biotype cluster c3^19^.

^d^Number of miRNA binding sites for miR-181, 188, 199 and 23 as defined in^15^ or using miRDB^87^.

**Figure 1:**
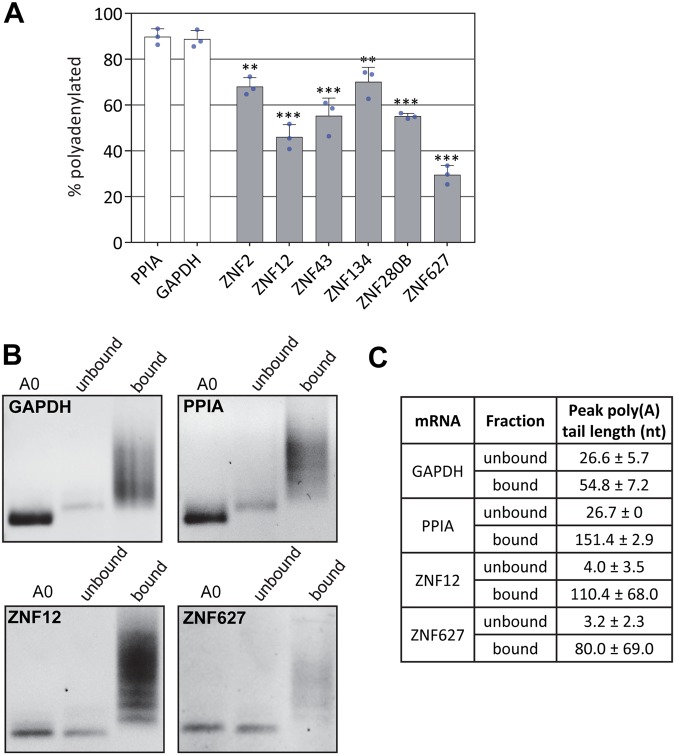
C2H2-ZNF mRNAs have a population with unusually short poly(A) tails. (**A**) RNA isolated from HeLa cells was fractionated by binding to oligo(dT) and the abundance of the indicated transcripts in each fraction was determined by qRT-PCR. The percentage of each mRNA in the poly(A)+ fraction is shown. Asterisks indicate significant differences from both PPIA and GAPDH controls (*p < 0.05, **p < 0.01, ***p < 0.001 one-way ANOVA, Tukey post-hoc test). Error bars represent standard deviations, blue circles represent individual data points. (**B**) RNA fractionated as described in A was assessed by LLM-PAT. The RNA used as template in the lane labeled 0 was treated with oligo(dT) and RNAse H prior to the assay to allow assessment of poly(A) tail length. (**C**) Poly(A) tail length was estimated from images like those shown in (**B**), using densitometry and comparison with a molecular weight marker. The errors are SEM derived from three independent experiments. Uncropped gels with markers are shown in Fig. S2.

First, we assessed whether the selected transcripts exhibited bimorphic behavior with respect to binding oligo(dT). Total RNA isolated from HeLa cells was fractionated using oligo(dT) magnetic beads and the proportion of each mRNA in each fraction was quantified by qRT-PCR. Control transcripts with poly(A) tails of known length were spiked in and evaluated in the same way (Fig. S1). As shown in Fig. 1A, PPIA and GAPDH, abundant polyadenylated mRNAs, were found almost exclusively in the bound fraction. However, all six of the C2H2-ZNF mRNAs had significant populations that failed to bind oligo(dT). Interestingly, this phenomenon is also observed in primary fibroblasts and in induced pluripotent stem cells (Fig. S3) suggesting it is not unique to cancer cells.

### C2H2-ZNF mRNAs can persist without a poly(A) tail

Failure of a fraction of mRNAs to bind oligo(dT) could either reflect a heterogeneous mixture of mRNAs with long and short poly(A) tails, or there may be a homogenous population having a poly(A) tail of an intermediate length (~12–25 nt) that binds inefficiently to oligo(dT). In order to distinguish these possibilities we used a qualitative RT-PCR-based assay^35^ to evaluate the poly(A) tail length of GAPDH, PPIA, ZNF12 and ZNF627 mRNAs in the oligo(dT) bound and flow-through fractions (Fig. 1B). The bound GAPDH and PPIA transcripts had a range of poly(A) tail lengths between ~30–250 nt in length, while those found in the unbound fraction all had tails of ~27 nt (Fig. 1C). This is consistent with observations that PABPC1 protects around 23–27 adenosine residues and that most mRNAs are rapidly degraded once the poly(A) tail is shortened to the point where PABPC1 can no longer associate. Thus, no transcripts completely lacking a poly(A) tail are detected^36–38^. Both ZNF12 and ZNF627 also have a range of tail lengths in the bound fraction, supporting that at least some of each population undergoes normal polyadenylation. However, in the major fraction that does not bind oligo(dT) these mRNAs exhibit strikingly little adenylation. The transcripts detected have very short, if any, poly(A) tails (Fig. 1B,C). This suggests that ZNF12 and ZNF627 mRNAs are relatively stable even when they lack the ability to associate with poly(A) binding proteins. Based on the results shown in Fig. 1A–C, we conclude that at least half the mRNA population for ZNF12 and ZNF627 has a poly(A) tail of less than 10 nt.

### C2H2-ZNF mRNAs are retained in the nucleus

**Figure 2:**
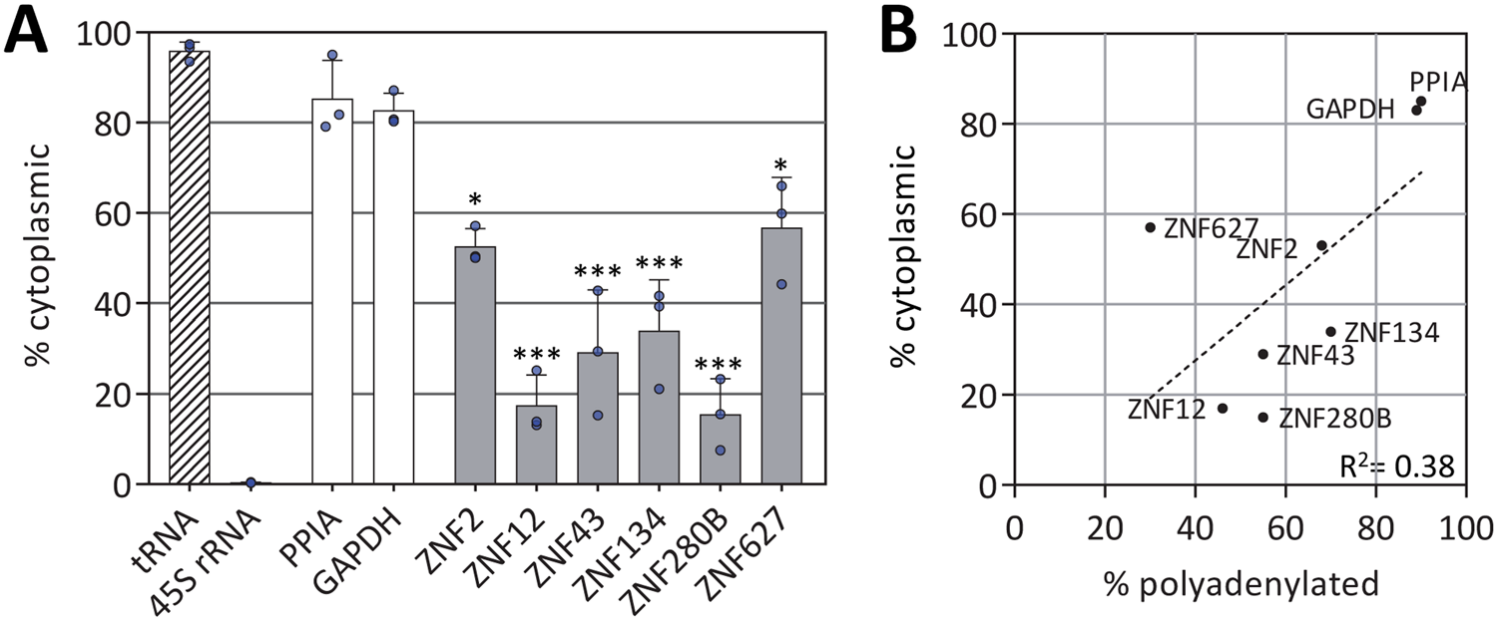
C2H2-ZNF mRNAs are restricted to the nucleus. (**A**) RNA was isolated from nuclear and cytoplasmic fractions of HeLa cells and the abundance of the indicated transcripts in each fraction was determined by qRT-PCR. The percentage of each mRNA in the cytoplasmic fraction is shown. Asterisks indicate significant differences from the PPIA and GAPDH controls (*p < 0.05, ***p < 0.001 one-way ANOVA, Tukey post-hoc test). Error bars represent standard deviations. Blue circles denote independent data points. (**B**) The percentage of each transcript in the cytoplasm was plotted against the percentage polyadenylated for six C2H2-ZNF mRNAs and the two control transcripts.

Polyadenylation is required for export of mRNAs, as well as influencing other aspects of mRNA metabolism^39^. Moreover, KRAB-domain ZNF mRNAs were enriched among mRNAs with a profile that includes increased nuclear retention^19,40^. We therefore wondered whether these ZNF transcripts with short poly(A) tails were also retained in the nucleus. We isolated nuclear and cytoplasmic RNA populations and quantified the level of ZNF and control RNAs in each fraction by qRT-PCR (Fig. 2A). Interestingly, while GAPDH and PPIA mRNAs are primarily cytoplasmic, all the ZNF transcripts had significantly larger populations retained in the nucleus. There is a clear correlation between the extent of polyadenylation and the proportion of the population exported to the cytoplasm (Fig. 2B). Nuclear retention of ZNF transcripts was also observed in fibroblasts and iPS cells (Fig. S4).

In order to address directly whether the nuclear retained transcripts also have short poly(A) tails we performed a two-step fractionation, in which we separated the nucleus and cytoplasm, isolated total RNA from each fraction and finally bound the RNA to oligo(dT) to separate polyadenylated RNAs. We quantified the amount of mRNA in each fraction by qRT-PCR as before. The results shown in Fig. S5 suggest that for five of the six ZNF mRNA the nuclear population generally exhibits slightly less polyadenylation than the cytoplasmic population. This difference is most obvious for ZNF43 and ZNF134 mRNAs where the cytoplasmic population is polyadenylated to a similar extent as GAPDH mRNA while the nuclear population is significantly less polyadenylated. This observation is consistent with the two phenotypes, nuclear retention and short poly(A) tails, being connected. However, as polyadenylated ZNF mRNAs are present in the nucleus and unadenylated ZNF mRNAs are detected in the cytoplasm we cannot draw any definitive conclusions.

### Sequence elements encoding C2H2 zinc fingers influence polyadenylation and export

**Figure 3:**
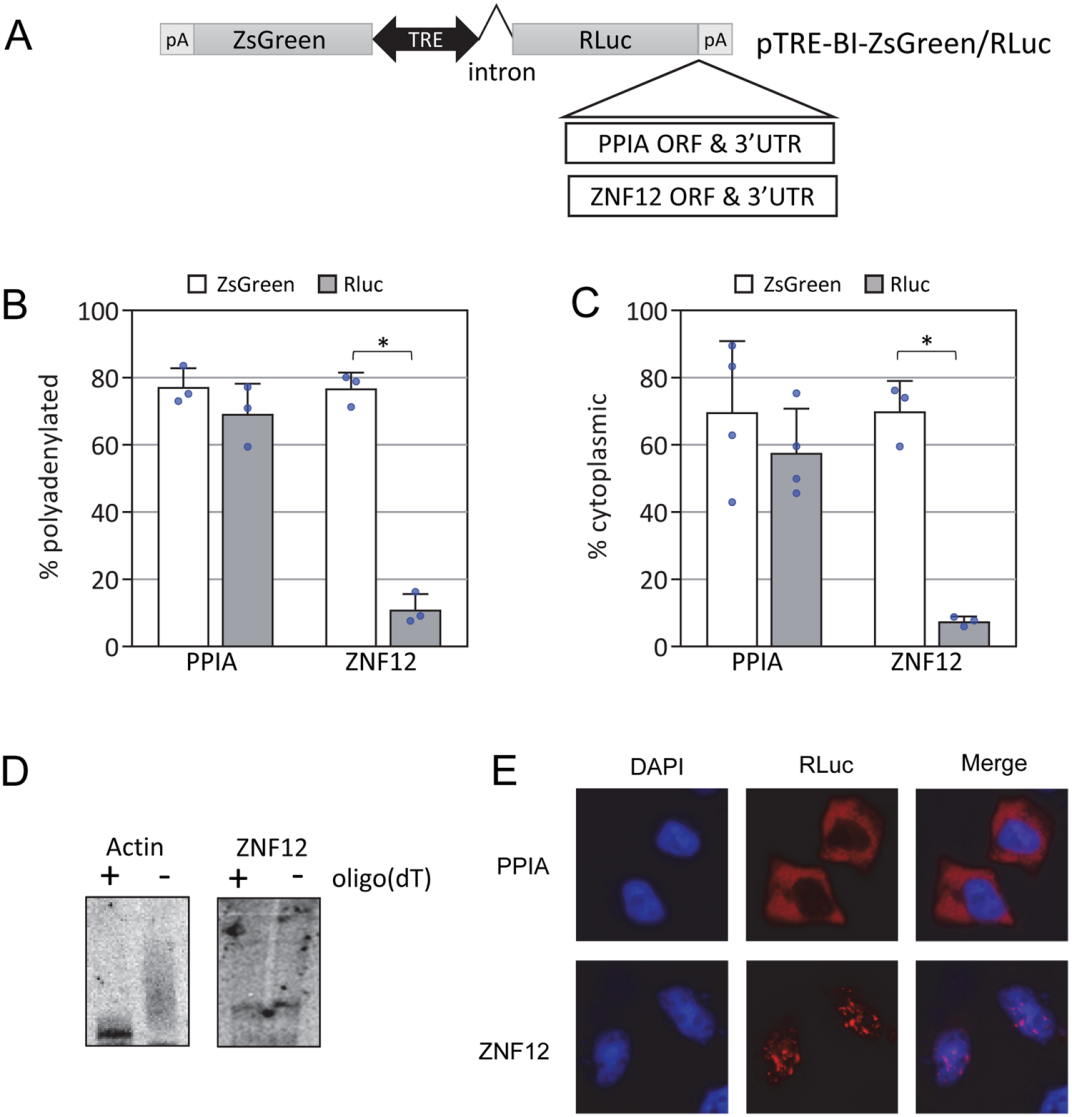
A reporter transcript bearing the ORF and 3′UTR of ZNF12 behaves like the endogenous transcript. (**A**) Cartoon depicting luciferase reporter constructs. The ORF and 3′UTR of ZNF12 or PPIA were cloned into pTRE-BI-ZsGreen downstream of RLuc. (**B**) The indicated reporter construct was transfected into HeLa Tet-Off Advanced cells. RNA was isolated and fractionated by binding to oligo(dT). The amount of the Renilla Luciferase and ZsGreen mRNA in each fraction was determined by qRT-PCR. *Denotes p < 0.05 two-tailed paired t-test. Error bars represent standard deviations. Blue circles denote individual replicate data points. (**C**) The indicated reporter construct was transfected into HeLa Tet-Off Advanced cells and nucleus and cytoplasm were separated. RNA was isolated and fractionated and the amount of the Renilla Luciferase and ZsGreen mRNA in each fraction was determined by qRT-PCR. *Denotes p < 0.05 two-tailed paired t-test. Error bars represent standard deviations. Blue circles denote individual replicate data points. (**D**) RNA isolated from HeLa Tet-OFF cells transfected with the ZNF12 reporter was treated with RNAseH and an oligonucleotide targeting the 3′UTR of Actin (Left) or ZNF12 (right). In lanes marked “+”, oligo(dT)18 was added during the RNAseH treatment to remove any poly(A) tail. RNA was separated on a denaturing polyacrylamide gel, blotted and detected using a 5′ end labeled oligonucleotide probe. (**E**) The indicated reporter construct was transfected into HeLa Tet-Off Advanced cells. Cells were fixed and the Renilla Luciferase mRNA was detected by fluorescence *in situ* hybridization. The nucleus was visualized by staining with DAPI.

In order to further characterize the mechanisms responsible for restricting the length of the poly(A) tail and retaining these transcripts in the nucleus, we created a reporter constructs in which the ORF and 3′UTR of the ZNF12 mRNA were fused to Renilla Luciferase with an upstream intron and expressed under a tetracycline responsive promoter (Fig. 3A). The same promoter was used to drive expression of ZsGreen in the opposite direction as an internal control for transfection efficiency. As a negative control, we created a similar reporter with the ORF and 3′UTR of PPIA. The ZsGreen mRNA behaved as expected and was primarily polyadenylated (Fig. 3B). Both the PPIA and ZNF12 reporter RNAs also behaved similarly to their endogenous counterparts despite having heterologous 3′ end formation signals (derived from the SV40 late poly(A) signal).

The PPIA reporter was primarily polyadenylated and cytoplasmic, while the ZNF12 reporter largely failed to bind oligo(dT) and was present mainly in the nuclear fraction (Fig. 3B,C). We also evaluated the poly(A) tail of the ZNF12 reporter via RNAseH northern blotting and found that, like the endogenous ZNF12 transcript (Fig. 1B), the vast majority of mRNA produced lacks poly(A) (Fig. 3D, Supplementary Fig. S6).Taken together these results suggest that sequence elements present in the 3′UTR and/or ORF of ZNF12 can confer nuclear retention, and restrict the length of the poly(A) tail. Conversely, the 5′UTR, the poly(A) signal and downstream sequences are not involved.

We next utilized RNA-FISH to assess the subcellular localization of each reporter. As expected, the PPIA reporter was primarily cytoplasmic with minimal nuclear accumulation. In contrast, the ZNF12 reporter was distributed throughout the cell with significant nuclear staining. This pattern is consistent with the distribution of the reporter and endogenous ZNF12 between nuclear and cytoplasmic fractions and supports that sequences in the ORF and/or 3′UTR of ZNF12 can influence the localization of the reporter transcript. Interestingly, most cells exhibited accumulation of the reporter RNA in multiple nuclear foci (Fig. 3E).

**Figure 4:**
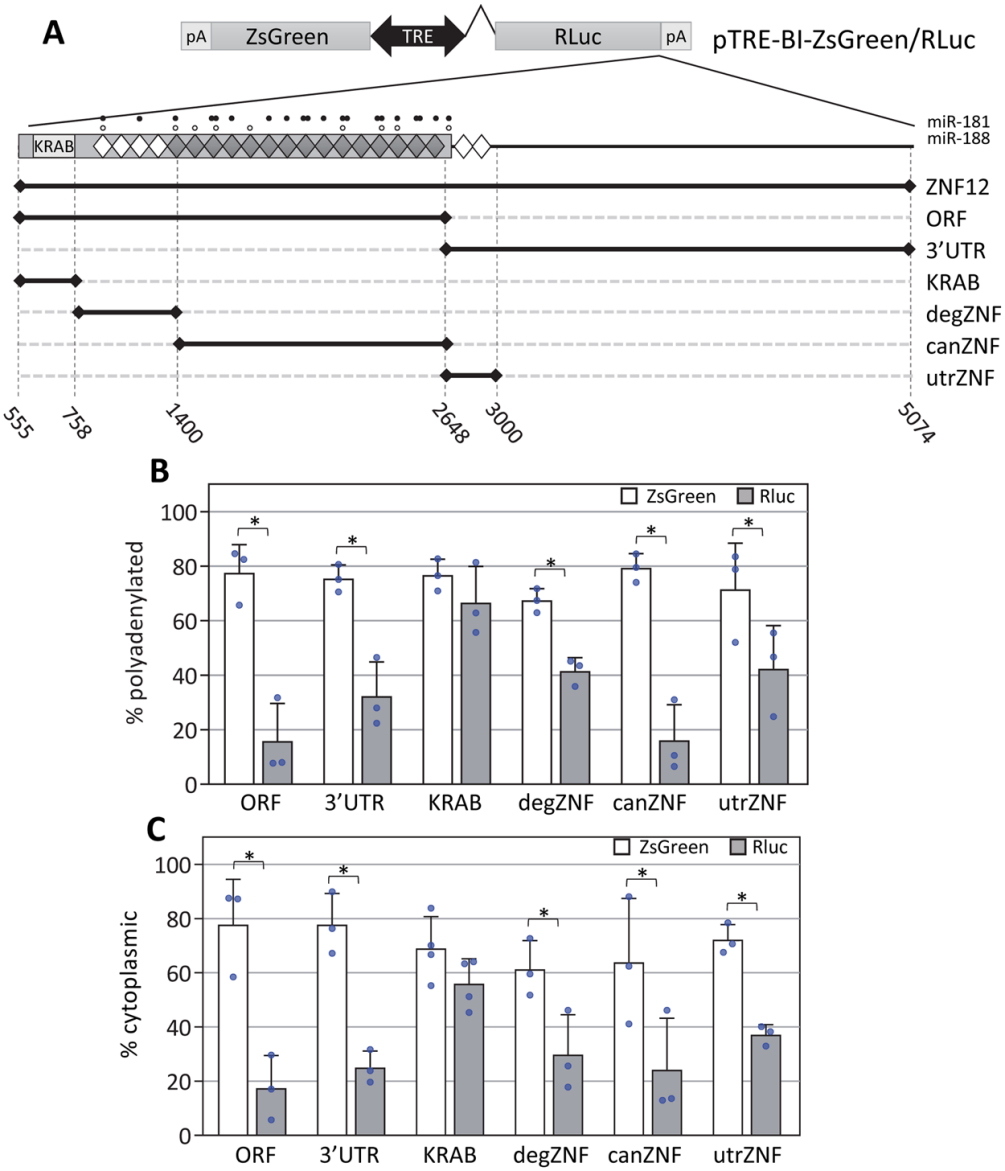
Sequences encoding canonical or degenerate C2H2-ZNF motifs confer short poly(A) tails and nuclear localization onto reporter transcripts. Several constructs were generated as indicated. Features of the ORF are depicted as follows: Filled rectangle = ZNF12 ORF, Filled and open circles = miR-181 and 188 binding sites, filled diamond = canonical zinc finger motif, open diamond = degenerate zinc finger motif, KRAB = Kruppel Associated Box. The constructs shown in A were transfected into HeLa Tet-Off Advanced cells and their poly(A) status (B) and localization (C) were assessed as described in Fig. 3. *Denotes p-value < 0.05 by two-tailed paired t-test. Error bars are standard deviations. Blue circles are data points derived from independent replicates.

In order to narrow down the region of the ZNF12 mRNA required for nuclear retention and poly(A) restriction, we cloned the ZNF12 ORF and 3′UTR separately into the RLuc reporter (Fig. 4A). Surprisingly, both of these constructs behaved similarly to the full-length reporter. These reporter mRNAs were predominantly nuclear and had a large population that failed to bind oligo(dT). The simplest explanation is that there are multiple redundant sequence elements in the 3′UTR and ORF that can influence poly(A) status and localization.

We hypothesized that perhaps the ZNF12 3′UTR might contain sequence elements encoding remnants of C2H2-ZNF motifs that were inactivated during gene duplication or other evolutionary events. This is a relatively common occurrence for C2H2-ZNF genes^41^. By aligning the ORF and 3′UTR to each other, we were able to identify ZNF-related sequence elements at the 5′ end of the 3′UTR as well as additional inactivated ZNF-related motifs within the 5′ half of the ORF closer to the KRAB domain (Fig. S7). These degenerate ZNF sequences are depicted as open diamonds in Fig. 4A. Thus, every construct tested to this point bears multiple sequence elements related to those encoding C2H2-ZNFs. This was strong evidence indeed that sequences encoding C2H2-ZNFs are responsible for the effects on polyadenylation and localization. We made three additional ORF constructs; one containing just the KRAB domain and no C2H2-ZNF sequences (KRAB), a second containing the degenerate ZNF motifs within the 5′ end of the ORF (degZNF) and the third containing the remaining canonical ZNF motifs (canZNF). We also made a construct bearing just the region of the 3′UTR that contains sequences that may have encoded functional C2H2-ZNF motifs in the past (utrZNF). As predicted, the KRAB only reporter behaved much like the PPIA control; the transcript was polyadenylated and exported to the cytoplasm. In contrast, all the reporters bearing ZNF-like sequences (degZNF, canZNF, utrZNF) behaved more like the full length reporter in that these mRNAs were predominantly nuclear and did not efficiently bind oligo(dT). There was a clear positive correlation between the level of polyadenylation and the cytoplasmic accumulation of the ZNF12 reporter constructs (Fig. S7B). We conclude that the sequences encoding ZNF motifs can influence poly(A) tail length and export from the nucleus.

C-rich sequence elements were recently identified as nuclear retention signals in several long non-coding RNAs^42,43^. HnRNP K was implicated as a factor recognizing these signals and mediating their effects^43^,which is notable because hnRNP K has also been linked with reduced polyadenylation of the lncRNA NEAT1-1^44^ and more generally with 3′ end processing and transcription termination^45^. It seemed conceivable that hnRNP K might be responsible for both the short poly(A) tail on ZNF mRNAs and their nuclear retention. There are short C-rich stretches in the Zinc Finger Coding (ZFC) regions that could possibly recruit hnRNP K (Fig. S6) but we could find no evidence in existing eCLIP datasets to suggest that hnRNP K associates with the ZFC regions of our ZNF mRNAs^46^. Nevertheless, as the connections between hnRNP K and both polyadenylation and nuclear retention were intriguing, we evaluated the effect of depleting hnRNP K on ZNF mRNA localization (Fig. S8). Unfortunately, despite efficient (>70%) knockdown of hnRNP K at both the mRNA and protein level (Fig. S7A,B), there was no discernable effect on ZNF mRNA localization or that of another nuclear-retained mRNA, MLXPIL, which was reported previously to undergo export following hnRNP K knockdown (Fig. S8D)^43^. We found that PTOV1-AS1, a lncRNA whose accumulation is dependent on hnRNP K^47^, was down-regulated (Fig. S7C) supporting that hnRNP K was sufficiently depleted to have a biological impact. Based on our results, and a dearth of evidence for association of hnRNP K with ZNF mRNAs, we conclude that hnRNP K is not likely to be a major component of the pathway responsible for nuclear retention of ZNF mRNAs.

### C2H2-ZNF mRNAs fail to experience polyadenylation

**Figure 5:**
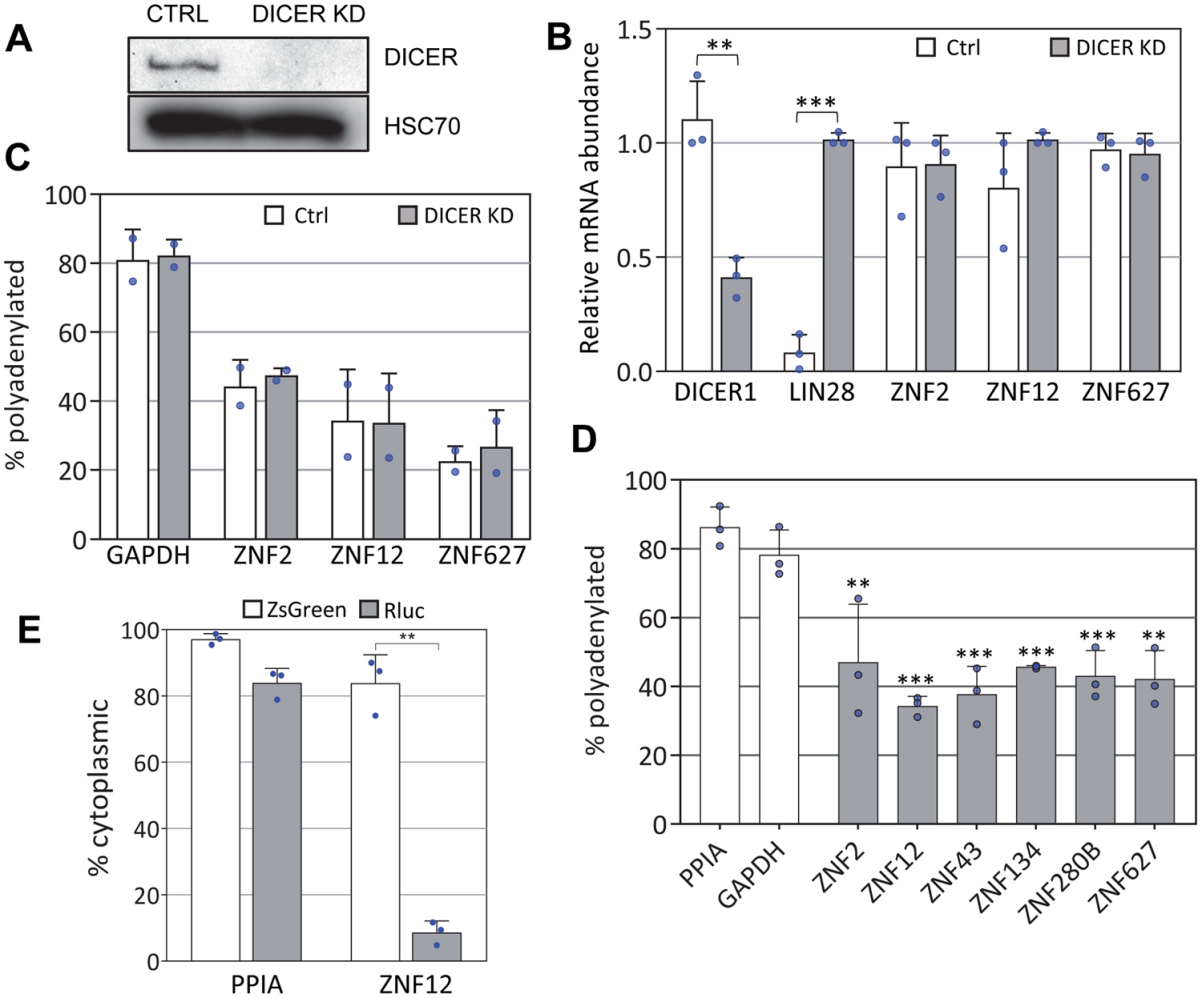
Neither DICER or polyadenylation are required for nuclear retention of ZNF mRNAs. (**A**) Western blot showing effective depletion of DICER protein in HeLa cells stably expressing an shRNA targeting DICER or empty vector. HSC70 was utilized as a loading control. Uncropped western blots are shown in Supplementary Fig. S10. (**B**) qRT-PCR analysis of mRNA abundances in CTRL and DICER KD cell lines. *Denotes p < 0.05 by two-tailed t-test. (**C**) qRT-PCR analysis following oligo(dT) fractionation of RNA from CTRL and DICER KD cell lines. (**D**) qRT-PCR analysis of mRNA abundances following oligo(dT) fractionation of nascent 4sU-labeled mRNAs. Asterisks indicate significant differences from both PPIA and GAPDH controls (**p < 0.01 ***p < 0.001 one-way ANOVA, Tukey post-hoc test). (**E**) The poly(A) signal of the ZNF12 and PPIA reporters was replaced with a DNA fragment from the 3′ end of the MALAT1 gene and nuclear/cytoplasmic distribution of each reporter was evaluated. **p < 0.01 one-way ANOVA, Tukey post-hoc test. For all panels, error bars represent standard deviations. Blue circles denote individual replicate data points.

Thus far, we have provided compelling evidence that the ~84 nt sequence that encodes ZNF motifs can restrict polyadenylation and lead to retention of ZNF mRNAs in the nucleus. This function is apparently independent of the ZNF12 poly(A) signal itself, as the majority of our constructs contain the SV40 late poly(A) signal. As the sequences coding for ZNF motifs are targeted by miRNAs^15,16^ we hypothesized that perhaps this interaction was contributing to the unusual metabolism of these mRNAs. If this were the case, then depletion of DICER (the enzyme that processes miRNAs in the cytoplasm) and the consequent reduction in miRNA levels should allow polyadenylation and export to occur. Despite strong evidence for expression of ZNF-targeting miRNAs in HeLa cells^48^ (Fig. S9), depleting DICER had no discernable effect on abundance, or polyadenylation of the ZNF transcripts (Fig. 5) although it did very effectively induce expression of LIN28A, a transcript repressed by the let-7 miRNA^49^. We conclude that interaction of miRNAs with the sequences encoding ZNF motifs does not influence poly(A) tail length of the ZNF mRNAs. This is consistent with the fact that the 3′UTR and utrZNF reporter mRNAs, which lack binding sites for ZNF-targeting miRNAs, still have short poly(A) tails and are retained in the nucleus (Fig. 4).

To determine whether ZNF mRNAs with very short tails fail to get polyadenylated immediately following 3′ end cleavage, or instead get polyadenylated efficiently and then deadenylated at a later time, we investigated the length of tail on nascent mRNAs. HeLa cells were treated with 4-thiouridine (4sU) for 10 minutes and then total RNA was isolated^50,51^. 4sU-labeled nascent RNAs were conjugated to biotin and isolated by binding to streptavidin beads. The eluted RNA was then bound to oligo(dT) beads as before, and ZNF mRNAs were assessed in each fraction by qRT-PCR. As shown in Fig. 5D, a large fraction (~50% or more) of the nascent ZNF transcripts, but not the PPIA and GAPDH control mRNAs, fail to bind oligo(dT) suggesting that the polyadenylation process itself may be ineffective for these mRNAs. It seems that this is a stochastic phenomenon as the remainder of the population binds oligo(dT), suggesting it is polyadenylated normally.

We wondered whether the polyadenylation machinery, or factors recruited following a failure to polyadenylate, might be responsible for nuclear retention of the ZNF transcripts. To investigate this idea we wanted to evaluate reporters with 3′ ends made independent of the polyadenylation machinery. The MALAT1 non-coding RNA 3′ end is formed through cleavage by RNAse P without involvement of the canonical polyadenylation machinery^52^. Heterologous transcripts bearing the MALAT1 3′ end signal are neither adenylated nor retained in the nucleus^53^. When we replaced the 3′ end of the full length ZNF12 and PPIA reporters with the MALAT1 3′ end signal, the PPIA reporter transcript was still efficiently exported but the ZNF12 reporter mRNA was retained in the nucleus (Fig. 5E) to a similar extent as the reporter bearing the SV40 poly(A) signal (Fig. 3C). Therefore, nuclear retention of the ZNF12 transcript is not due to aberrant or failed polyadenylation, although we cannot at this point rule out that the lack of a poly(A) tail is significant.

## Discussion

In the experiments described above, we have shown that C2H2-ZNF mRNAs can be synthesized as two populations; with long/normal and very short poly(A) tails. In addition, these mRNAs exhibit a nuclear distribution consistent with failure to export, or with very rapid decay upon entry to the cytoplasm. There is a clear correlation between the level of polyadenylation and the efficiency of export for C2H2-ZNF mRNAs and the same sequence elements seem to influence both phenomena. Notably, both failure to polyadenylate and nuclear retention are conferred on a heterologous RNA by sequences capable of encoding C2H2 zinc finger motifs, which we have called Zinc finger Coding Regions or ZCRs. Furthermore, ZNF12 reporter mRNAs that do not experience polyadenylation are also retained in the nucleus supporting that nuclear retention can be separated from the polyadenylation process.

### Possible mechanisms for poly(A) tail length restriction

Polyadenylation initiates with recognition of the poly(A) signal (AAUAAA) upstream of the cleavage site and a downstream element (DSE) by Cleavage Polyadenylation Specificity Factor (CPSF) and Cleavage Stimulation Factor (CstF) respectively. Following cleavage, poly(A) polymerase (PAP) begins to add adenosine residues to the 3′ end of the mRNA^54^. Once the poly(A) tail becomes long enough to bind the nuclear poly(A) binding protein (PABPN1), an interaction between PAP, CPSF and PABPN1 causes the polyadenylation reaction to become more processive and the tail is rapidly extended to ~250 nt^24^. The C2H2-ZNF mRNAs appear to undergo cleavage, but the poly(A) tail is not extended beyond a few nucleotides suggesting that recruitment or activity of PAP may be impaired by factors associating with the ZCR. Alternatively, ZCR-binding factors could prevent recruitment of PABPN1 or other factors connected with poly(A) tail length control such as ZC3H14^55^.

One characterized mechanism to inhibit polyadenylation is through binding of splicing factors that can inhibit PAP close to the poly(A) site^56,57^. However, this phenomenon requires binding of the inhibitory factor relatively close to the poly(A) signal while ZCRs are able to function from a distance. In addition, inhibition of polyadenylation by splicing factors generally results in reduced mRNA abundance suggesting that the unadenylated transcripts are degraded but the C2H2-ZNF mRNAs are clearly able to persist despite their short poly(A) tails. ZCRs bear more functional similarity to poly(A) limiting elements (PLE) which can be located in the coding region, within the last exon, and can restrict polyadenylation even in the presence of a heterologous strong poly(A) signal^31^. However, unlike the C2H2-ZNF mRNAs, PLE-containing mRNAs appear to be exported and translated efficiently despite lacking a poly(A) tail^32^. The mechanism by which PLEs limit polyadenylation is not understood, although the splicing factor U2AF65 associates with PLEs and can modulate their activity^58^. Notably, when we prevented polyadenylation, by replacing the poly(A) signal with the MALAT1 3′ end sequence, the ZNF12 reporter mRNA was still retained in the nucleus suggesting that any *trans*-acting factors involved in nuclear retention are not recruited by the polyadenylation machinery.

### Nuclear retention of RNAs

It appears there are many mechanisms for RNAs to be retained in the nucleus. In some cases, a specific and rather short sequence element (such as the C-rich SIRLOIN element in Alu containing mRNAs^43^ or the AGCCC element in BORG^59^) is sufficient to prevent export by recruiting a protein factor such as hnRNP K. Another protein able to block RNA export is the ZFC3H1 exosome adaptor which can compete with the export factor ALYREF for binding to the RNA^60^. In the case of lncRNAs like MALAT1 and NEAT1, the minimal region required for nuclear retention is much longer (100 s of nucleotides) and may function by providing structural scaffold for proteins to bind and/or regions for RNA-RNA hybridization^61^. It appears specific properties of these lncRNAs allow them to nucleate phase separation and accumulate in subnuclear compartments^62^. Given that the ZNF12 reporter accumulates in foci (Fig. 3E) and the ZFCs have no known nuclear retention sequence in common, they may be functioning in a similar way to lncRNAs.

Nuclear export requires assembly of an export-competent RNP which generally depends on accurate and complete processing of the transcript^63^. Failure to splice, cap or polyadenylate a nascent mRNA can lead to degradation or a delay in export due to formation of aberrant mRNPs that do not recruit the appropriate export factors. Transcripts that undergo non-canonical processing have alternative fates. For example, mRNAs lacking introns must rely on additional signals for export^64,65^. Importantly, the alternative 3′ end processing pathway experienced by MALAT1 results in a transcript with no poly(A) tail, but this is not sufficient on its own to result in nuclear retention: The MALAT1 3′ end cannot specify nuclear retention of the PPIA reporter (Fig. 5E) or of a GFP mRNA^53^. In order for an RNA bearing the MALAT1 3′ end to be retained in the nucleus, it requires some additional property conferred by a ~600 nt region of the MALAT1 RNA body^61^, or as shown here, by ZFCs. Importantly, the MALAT1 nuclear retention element can also function on an RNA bearing a canonical poly(A) signal, although it is not clear whether this transcript actually acquired a poly(A) tail^61^. Based on this result we propose that the ZFCs function through a pathway similar to that utilized by MALAT1. In this respect, we note many non-coding RNAs that undergo canonical splicing and polyadenylation, including the Xist^66^, Bsr^67^ and BORG^59^ lncRNAs, are retained in the nucleus through various mechanisms, and in a variety of subnuclear locations^68^. Certain factors binding the ZCR could perhaps reduce recruitment of export factors by partitioning these mRNAs to inaccessible nuclear domains or by preventing interactions between the export machinery and mRNA processing factors.

### Alu elements are unlikely to be involved

Transcripts containing inverted repeat (IR) *Alu* elements are retained in the nucleus, in paraspeckles, due to extensive post-transcriptional A-to-I editing^69^. *Alu* elements also contain C-rich sequences that drive nuclear localization through binding of hnRNP K^43^. Although C2H2-ZNF genes are more likely to contain *Alu*-derived sequences than other transcripts^23^, and C2H2-ZNF mRNAs are significantly enriched among a group of 333 genes with 3′UTR IR-*Alu* elements^70^, this mechanism cannot explain our observations for several reasons. First, only one of the six ZNF mRNAs we studied here (ZNF43) retains IR-*Alu* sequences in the mature transcript and experiences editing^70^. Second, unlike the C2H2-ZNF mRNAs, the vast majority of *Alu*-containing mRNAs are polyadenylated^17^. Third, there is no evidence for association of hnRNP K with the transcripts we evaluated^46^ and hnRNP K knockdown had no effect on localization of the ZNF mRNAs (Fig. S7). Finally, the nuclear retention of *Alu*-containing mRNAs is abrogated in embryonic stem cells concomitant with loss of paraspeckles^71^; yet the ZNF mRNAs we tested are retained in the nucleus even in pluripotent cells (Fig. S4) and C2H2-ZNF mRNAs were over-represented in the bimorphic population in H9 embryonic stem cells^17^.

### The nucleus as a storage compartment

The retention of C2H2-ZNF mRNAs within the nucleus presumably prevents them from functioning as mRNAs. Nuclear retention is an established mechanism to regulate gene expression. For example, the CTN non-coding RNA contains IR-*Alu* repeats that are extensively edited, leading to nuclear retention as described above. However, in response to stress, the CTN transcript is processed to remove the edited region and generate the mature mCAT2 mRNA^72^. The mCAT2 mRNA is efficiently exported and translationally competent. A more widespread pathway for delaying nuclear export is through retention of introns^73,74^. Transcripts with one or more unspliced introns can remain in the nucleus until an appropriate stimulus induces completion of splicing and allows formation of an export competent RNP^74,75^. Although our reporter transcripts have an intron and undergo splicing, the ZFC element can still retain RNAs within the nucleus. Further investigation is required to determine whether a poly(A) tail or the polyadenylation experience, can overcome this retention. If this were the case, then polyadenylation could perhaps induce rapid export and translation when needed, such as during certain phases of the cell cycle, or in response to stress.

### Sequence elements with dual coding and non-coding functions

There are several instances where coding region sequence elements moonlight to regulate mRNA export or other aspects of mRNA metabolism^76^. One particularly relevant example is the Signal Sequence Coding Region (SSCR) found at the 5′ end of transcripts encoding secretory proteins which both encodes signal peptide and enhances RNA export^77^. In this case, the SSCRs role as an mRNA export element does not compete with its role in coding for protein because the two functions are required sequentially. However, in other examples a non-coding function can compete with the coding function of an mRNA. This is the case for the bifunctional Steroid Receptor RNA Activator, SRA, which acts as an RNA scaffold to assemble factors involved in nuclear receptor signaling^78^. An alternatively spliced isoform of the SRA RNA encodes a protein that plays a role in trans-activation of nuclear steroid hormone receptors in the nucleus. The coding isoform is exported to the cytoplasm and translated and thus cannot act as a nuclear scaffold while the non-coding isoform lacks appropriate start codons, fails to be exported and cannot be translated to make protein^79^. It seems possible that the C2H2-ZNF mRNAs have acquired a nuclear non-coding function such as acting as a scaffold or miRNA decoy.

Future experiments will aim at characterizing the ZCRs in more detail, and identifying *trans-*acting factors that associate with the ZCRs to prevent polyadenylation and/or export. We are also interested in characterizing the nuclear domain these transcripts are accumulating in and evaluating whether cellular conditions such as stress or the cell cycle influence export and/or polyadenylation of these RNAs. Such studies might eventually allow us to modulate the polyadenylation/export of the entire class of C2H2-ZNF bimorphic transcripts and discern the biological impact.

## Methods

### Plasmid Constructs

In order to generate templates for *in vitro* transcription of control RNAs with different lengths of poly(A) tail, fragments of the *S. cerevisiae* ACT1, MET3 and MET25 genes were amplified by RT-PCR using primers shown in Table S1A and cloned between the *Bam*HI and *Sal*I sites of pGEM4 (Promega) plasmids that had adenosine stretches of 15, 45 or 149 adenosines, respectively, inserted 3′ of the multiple cloning site. These plasmids were linearized with *Pvu*II and the empty pGEM4 plasmid was linearized with *Sma*I (to generate an RNA with no poly(A) tail). Each linearized plasmid was *in vitro* transcribed using SP6 RNA polymerase (Thermo Fisher Scientific) and the resulting RNA was gel purified before use.

The parent vector for ZNF12 and PPIA expression constructs was generated by cloning the β-globin/IgG chimeric intron from pCI-Neo (Promega) into the *Apa*I site of pTRE3G-BI-ZsGreen (Clontech), and then inserting Renilla luciferase amplified from pLightSwitch (Switchgear Genomics) into the *Bgl*II/*Not*I sites. The empty parent vector was digested with *Not*1. Fragments containing ORF and/or 3′UTR sequences from ZNF12 (NM_016265.3) and PPIA (NM_021130.4) were amplified from HeLa cDNA or from previously generated plasmids. The 3′ end of the mouse MALAT1 gene (NR_002847.3) was synthesized as a gBlock (Integrated DNA Technologies) and used to replace the 3′ end of PPIA or ZNF12 in the original clones. Individual constructs were created using In-Fusion HD (Clontech) or NEBuilder HiFi (New England Biolabs) which are ligation independent cloning kits. Primers for the PCR reactions are shown in Table S1A. Fragments were combined with vector in the ratios recommended by the manufacturer and incubated at 50 °C for 20–30 min before transformation into *E. coli*. All plasmids were sequenced prior to use.

### Cell Culture and Transfection

HeLa S3 cells adapted to adherent culture were maintained in DMEM (4.5 g/L glucose) supplemented with 10% fetal bovine serum (FBS), 2 mM L-glutamine, and 0.37% sodium bicarbonate at 37 °C in 5% CO_2_. HeLa Tet-Off Advanced cells (Clontech) were maintained in DMEM supplemented with 10% Tetracycline-free FBS (Clontech), 2 mM L-glutamine, 0.37% sodium bicarbonate, and 100 μg/mL G418 to maintain expression of the transactivator.

Induced Pluripotent Stem (iPS) cells (System Biosciences Cat # SC101A-iPSC, Lot # 110415-01) were cultured on Matrigel (BD Biosciences) in mTesR1 media (STEMCELL Technologies) at 37 °C in 5% CO2. Human foreskin fibroblasts (HFFs; System Biosciences Cat # SC101A-HFF, Lot # 110509) were maintained in DMEM (4.5 g/L glucose) supplemented with 2 mM L-glutamine, 0.1 mM non-essential amino acids, and 10% FBS at 37 °C, 5% CO_2_.

HeLa Tet-Off Advanced cells were transfected with reporter plasmids using jetPRIME (Polyplus) prior to allowing them to adhere to the dish. A ratio of 3 µL reagent to 1 µg of plasmid was employed according to the manufacturer’s recommendations. Cells were harvested 24–48 hr after transfection. Transfections were evaluated for ZsGreen expression using a Nikon Diaphot 200 microscope with a GFP-B filter cube (Nikon). Transfection efficiency was generally >70% at ~48 hours post transfection.

### Nuclear/Cytoplasmic Fractionation

Nuclei and cytoplasm were separated as described previously^80^. Cells were scraped into ice cold phosphate buffered saline (PBS), collected by centrifugation, resuspended in NP40 lysis buffer (0.5% NP-40, 10 mM Tris-HCl pH 8.5, 1.5 mM MgCl2, 10 mM EDTA, 140 mM NaCl) and incubated on ice for 5 min. Nuclei were pelleted by centrifugation for 5 min at 500 × g at 4 °C, the supernatant/cytoplasm was transferred to a fresh tube and an equal volume of TRIzol was added. After washing with NP40 lysis buffer, the nuclear pellets were lysed in TRIzol. RNA was isolated as described below. The RNA from the nucleus and cytoplasm was resuspended in an equal volume such that an equal number of cells were represented in 1 µl regardless of RNA concentration.

### RNA Isolation and qRT-PCR

TRIzol was added to cells after removal of the media. RNA isolation was performed according to the manufacturer’s instructions except an additional phenol/chloroform/isoamyl alcohol (25:24:1) extraction was performed immediately prior to addition of isopropanol. Total RNA was treated with DNAse I (Thermo Fisher Scientific) or with TURBO DNAse (Thermo Fisher Scientific) and *Dpn*I restriction enzyme if plasmid DNA had been transfected into the cells. DNase was removed by phenol/chloroform/IAA extraction and ethanol precipitation.

Reverse transcription was performed in 20 µL reactions with Improm-II Reverse Transcriptase (Promega) using 0.5 µg of random hexamers, according to the manufacturer’s instructions. 1–2.5 µL of cDNA was used to set up triplicate qPCR reactions using IQ SYBR-Green Supermix (BioRad) and primers as listed in Supplementary Table 1. A standard 2 step amplification protocol was performed using a BioRad CFX96 instrument with annealing/extension at 60 °C for 30 sec and denaturation at 95 °C for 10 sec. Amplification efficiency was determined for all primer pairs (Suppl. Table 1) and each pair produced a single product of the expected molecular weight. Relative abundance of mRNAs was determined using the BioRad CFX Manager™ software which relies on the Pfaffl method^81^.

### Control RNAs and Oligo(dT) Selection

80 fmol of a mixture of *in vitro* transcribed control RNAs with poly(A) tails of 0, 15, 45 and 149 nt were spiked into 10–20 µg total RNA samples in binding buffer (20 mM Tris-HCl, pH 7.5, 500 mM LiCl, 0.5% LiDS, 1 mM EDTA, 5 mM DTT) prior to fractionation. Oligo(dT)_25_ magnetic beads (New England Biolabs) were equilibrated in binding buffer. RNA samples were denatured at 70 °C for 2 min before being mixed with the beads and incubated at room temperature for 10 min. A magnetic field was used to retrieve the beads and the unbound fraction was reserved. The beads were washed with wash buffer (20 mM Tris-HCl, pH 7.5, 500 mM LiCl,0.1% LiDS, 1 mM EDTA, 5 mM DTT) containing 1 µL Ribolock RNase Inhibitor (Thermo Fisher Scientific) and the wash was added to the unbound fraction. This step was repeated with wash buffer and again with low salt buffer (20 mM Tris-HCl, pH 7.5, 200 mM LiCl, 1 mM EDTA), with the flow through being added to the unbound fraction each time. Finally, the poly(A)^+^ RNA was eluted in Tris-HCl, pH 7.5, 1 mM EDTA at 50 °C for 2 min. This step was repeated. The RNA in each fraction was recovered by precipitation with glycogen carrier and resuspended in 20 µL of nuclease-free water. Equivalent volumes of the bound (poly(A)+) and unbound(poly(A)−) fractions were used to make cDNA.

### Labeling and Isolation of Nascent RNAs

HeLa cells were treated with 500 µM 4-thiouridine for 10 min and then scraped into TRIzol. RNA was isolated using the RNAeasy kit (Qiagen) according to the manufacturer’s protocol. 75 µg of total RNA was labeled with biotin and fractionated using streptavidin magnetic beads (Miltenyi Biotec) as described previously^50^.

### Linker-Ligation-Poly(A) Tail (LLM-PAT) Assay

The LLM-PAT assay was performed as described previously^35^. Briefly, a population with no poly(A) tail was generated by treatment with oligo(dT)_18_ and 5 U of RNAse H (Fischer-Fermentas, EN0201) for 30 minutes. Next, 1 µg of this treated sample and an untreated RNA sample were each ligated to a linker RNA (10 µM 5′rApp-TTTAACCGCGAATTCCAG-ddC-3′, Linker-3 Integrated DNA Technologies) using T4 RNA Ligase 1 (New England Biolabs) at 16 °C overnight. The RNA was recovered and converted to cDNA using a primer complementary to the linker (5′-CTGGAATTCGCGGTT-3′) and Improm II Reverse Transcriptase (Promega). Finally, the 3′ end of each transcript of interest was amplified by PCR using OneTaq Hot Start Polymerase (New England Biolabs) employing a gene-specific forward primer paired with the linker primer as the reverse primer. The annealing temperature (55–59 °C) and number of cycles (27–32) were adjusted to optimize the amount of product for easy visualization. PCR products were separated and visualized by agarose gel electrophoresis and imaged on a BioRad Gel Doc EZ Imager. Image Lab Software (BioRad) was used to determine poly(A) tail lengths.

### RNase H Northern Blot Analysis

HeLa Tet-Off Advanced cells were transfected with the ZNF12 reporter plasmid as described above. Cells were then collected in TRIzol and total RNA was prepared. 10 µg of total RNA was hybridized to a gene-specific DNA oligonucleotide (Actin [5′-GTGGACTTGGGAGAGGACTG-3′] or ZNF12 [5′-TATACAACTTACCAGATGTAATT-3′]) complementary to a region just upstream of the poly(A) signal. Oligo(dT) was included as indicated. Following treatment with 5 U of RNAse H (Fischer-Fermentas, EN0201) for 30 minutes, RNA was recovered and separated on a 5% denaturing polyacrylamide gel. RNA was then transferred to a nylon membrane (Hybond N+, RPN119B GE Healthcare) followed by UV crosslinking. The blots were then pre-washed (0.1xSSC/0.1%SDS) at 65 °C for 1 hour, pre-hybridized (10x Denhart’s Solution, 6x SSC, 0.1%SDS) at 42 °C for 1 hour and then probed using ^32^P-end-labeled DNA oligonucleotides (resuspended in pre-hybridization buffer) specific to the gene of interest (Actin [5′-CATAATTTACACGAAAGCAATGCTATC-3′] or ZNF12 [5′-CACAAATATGCTATGAATATAG-3′]) overnight. The following day, blots were washed (6xSSC/0.1%SDS) three times at room temperature for 5 minutes followed by an additional wash at 50 °C for 20 minutes. Following an overnight exposure, phosphor screens were scanned using a Typhoon Trio (GE Healthcare) and the results analyzed with ImageQuant software (GE Healthcare).

### DICER and HNRNPK knockdown and western blotting

For DICER knockdown, HeLa S3 cells were transduced with lentiviral particles containing DNA encoding an shRNA that targets DICER (Sigma-Aldrich:TRCN0000051261) or a negative control lentiviral particles generated using the pLKO-1 empty vector^82^. Pools of stably transduced cells were selected with 10 μg/mL puromycin and then maintained in 1 μg/mL puromycin.

To transiently knockdown HNRNPK, HeLa Tet-Off Advanced cells were transfected with a pre-designed siRNA targeting HNRNPK (Sigma, Mission SASI_Hs02_00333311, target sequence: 5′-GCAAGAATATTAAGGCTCT-3′) or control siRNA (Sigma SIC001) at a final concentration of 50 nM using jetPRIME (Polyplus) reagent in accordance with the manufacturer’s protocol. Knockdown was assessed 48 hours after transfection.

In each case, knockdown was verified by qRT-PCR using primers described in Table S2 and also by western blot using rabbit anti-DICER antibodies (H212; Santa Cruz Biotechnology; 1:50 dilution in Tris-buffered saline containing 0.1% Tween-20) or rabbit anti-HNRNPK antibodies (Abcam ab18195, 1:1000). As a loading control, HSC70 was detected using rat anti-HSC70 (1B5; Santa Cruz Biotechnology; 1:1000 dilution) or GAPDH was detected using mouse anti-GAPDH (Millipore Sigma, MAB374, 1:5000). Detection was accomplished using HRP-conjugated secondary antibodies and SuperSignal West Pico Chemiluminescent Substrate (Thermo Fisher Scientific). Blots were imaged using a ChemiDoc XRS (BioRad) and quantified using ImageLab (BioRad).

### RNA-fluorescent *in situ* hybridization (RNA-FISH)

HeLa Tet-Off Advanced cells were transfected with PPIA or ZNF12 reporters and plated on coverslips. After 24–30 hr, cells were fixed and permeabilized using 3:1 methanol:acetic acid for 10 min at room temperature and an additional 2 hr at 4 °C. After washing in Wash Buffer (10% deionized formamide in 2xSSC) the cells were treated with Stellaris RNA-FISH hybridization buffer containing 125 nM of Stellaris RNA-FISH Q570 probe targeting Renilla luciferase (VSMF-1034–5, Biosearch Technologies, Inc.) at 37 °C overnight. Coverslips were washed in Wash Buffer and then in 1xSSC before mounting with Prolong Diamond AntiFade mountant with DAPI (Thermo Fisher Scientific). The cells were visualized using an Olympus IX71 inverted fluorescent microscope at 100X magnification using the 31000 DAPI/Hoechst filter (EX360, EM460) to visualize DAPI and the 41002 TRITC (Rhodamine)/Cy3 filter (EX535, EM610) to visualize Quasar570. Images were captured using Q Imaging Retiga 2000R camera.

### Data analysis

To determine the percentage of an mRNA bound to oligo(dT) the amount of each mRNA in equal volumes of the bound and flow through fraction was assessed by qRT-PCR and added to give the total. For graphing and subsequent analysis the amount bound to oligo(dT) was expressed as a percentage of this total. Similarly, to determine the percentage of an mRNA in the cytoplasm the amount of each mRNA in equal volumes of the cytoplasmic and nuclear fractions was assessed by qRT-PCR and added to give the total. The amount in the cytoplasm was expressed as a percentage of the total for graphing and analysis.

Three independent replicates were performed for each experiment unless otherwise noted. For pairwise comparisons, a paired two-tailed t-test was employed. For multiple comparisons, Levene’s test^83^ was used to demonstrate equal variance, then a one-way ANOVA test was performed with post-hoc Tukey test^84^ or Dunnett’s test as indicated. P values of less than 0.05 were considered significant.

Much of the work described was published in a thesis by Aimee L. Jalkanen^85^.

## Electronic supplementary material

Supplemental Figures
