# Supplementary material for: Sequences encoding C2H2 zinc fingers inhibit polyadenylation and mRNA export in human cells
Source: Sci Rep. 2018 Nov 19;8:16995. doi: 10.1038/s41598-018-35138-4 (PMC6242934; doi:10.1038/s41598-018-35138-4)
Supplement: Supplementary file 1 — Supplemental Figures [file 41598_2018_35138_MOESM1_ESM.pdf]

Supplementary Figures 1-10

Sequences encoding C2H2 zinc fingers inhibit polyadenylation and mRNA export in human cells

Joseph Russo, Aimee L. Jalkanen, Adam M. Heck, Caleb M. Schmidt, Jeffrey Wilusz, Carol J Wilusz

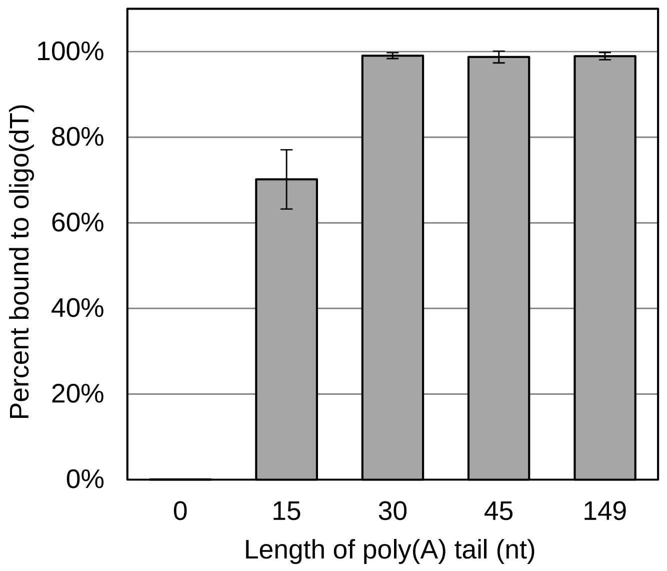

Supplementary Figure 1: *In vitro* transcribed RNAs with poly(A) tails of 30 nucleotides or greater are bound effectively to oligo(dT)$_{18}$ magnetic beads. Error bars represent the standard deviation from 3 replicates.

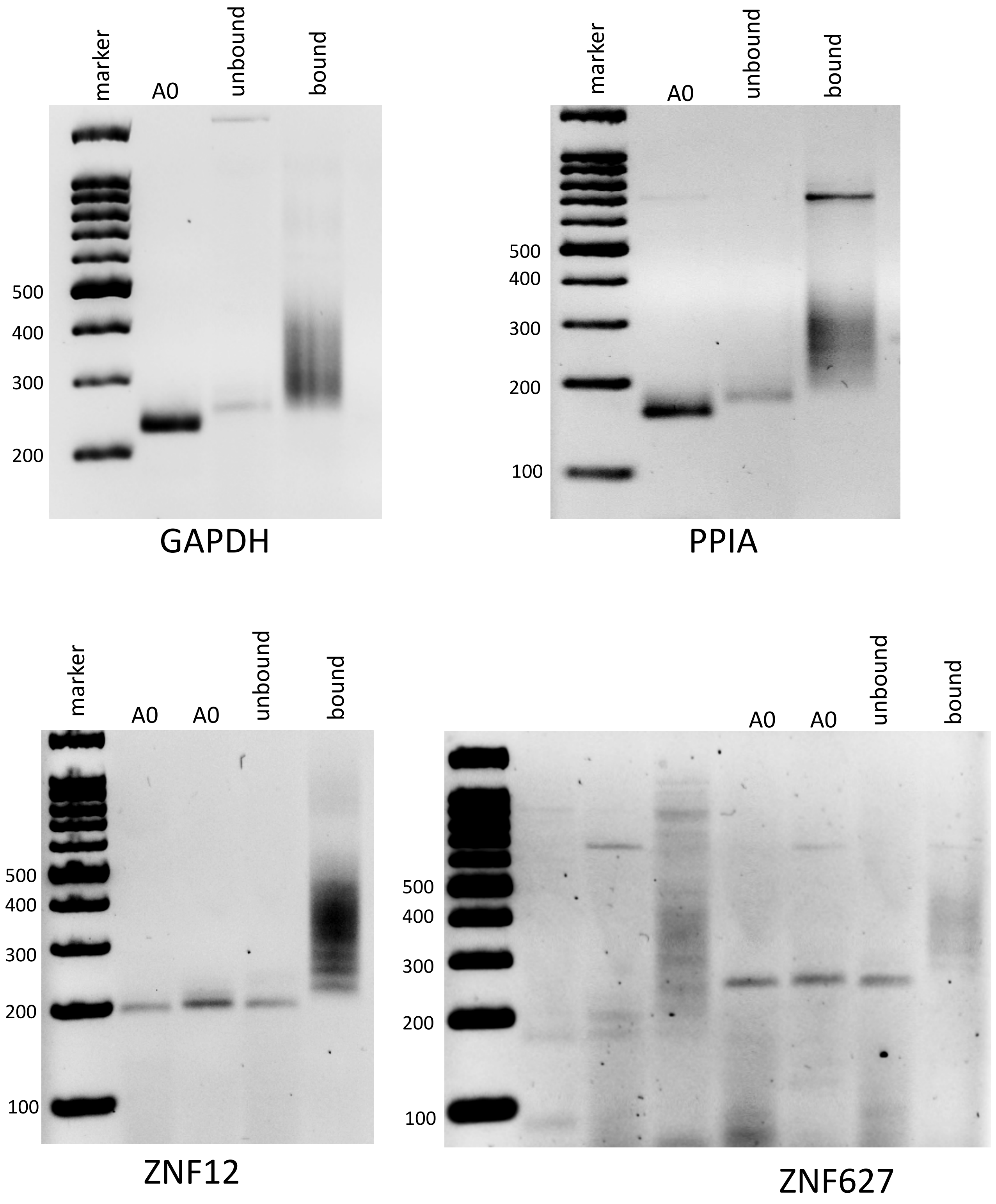

Supplementary Figure 2: Uncropped images for LLM-PAT assays shown in Figure 1B.

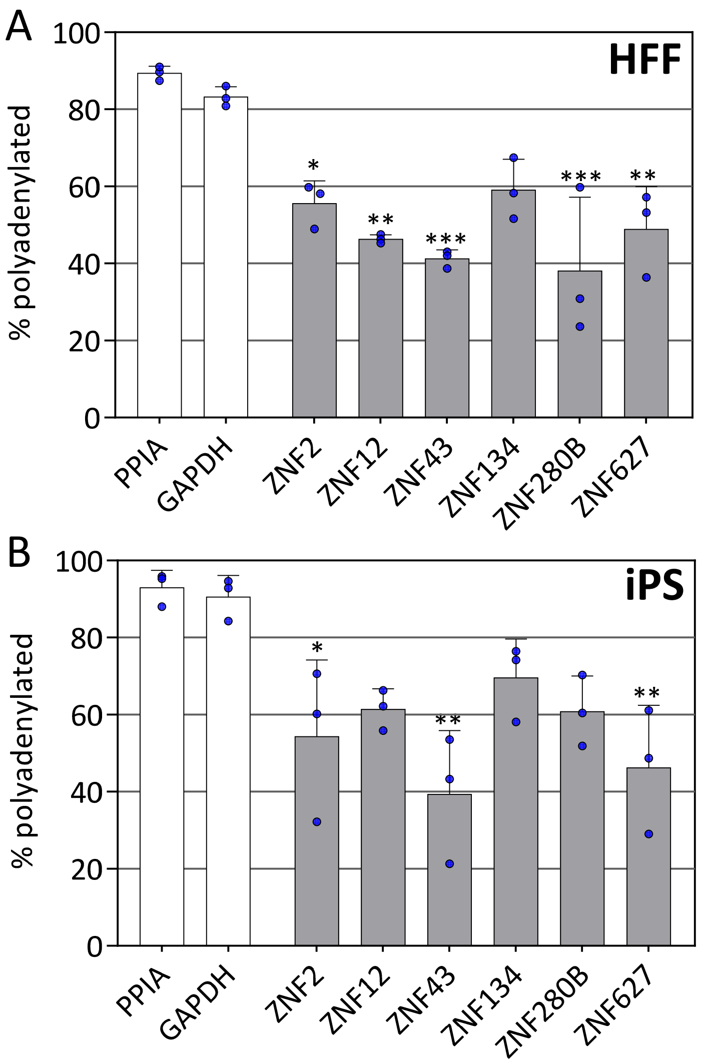

Supplementary Figure 3. C2H2-ZNF mRNAs have short poly(A) tails in normal cell lines. RNA isolated from HFF (A) or iPS (B) cells was fractionated by binding to oligo(dT) and the abundance of the indicated transcripts in each fraction was determined by qRT-PCR. The percentage of each mRNA in the poly(A)+ fraction is shown. Blue dots indicate independent replicates. Error bars represent standard deviations. Asterisks indicate significant differences from both PPIA and GAPDH controls (*$p<0.05$, **$p<0.01$, ***$p<0.001$ one-way ANOVA, Tukey post-hoc test). ZNF134 was significantly different from PPIA ($p<0.05$) but not from GAPDH in the HFF cell line.

Supplementary Figure 4

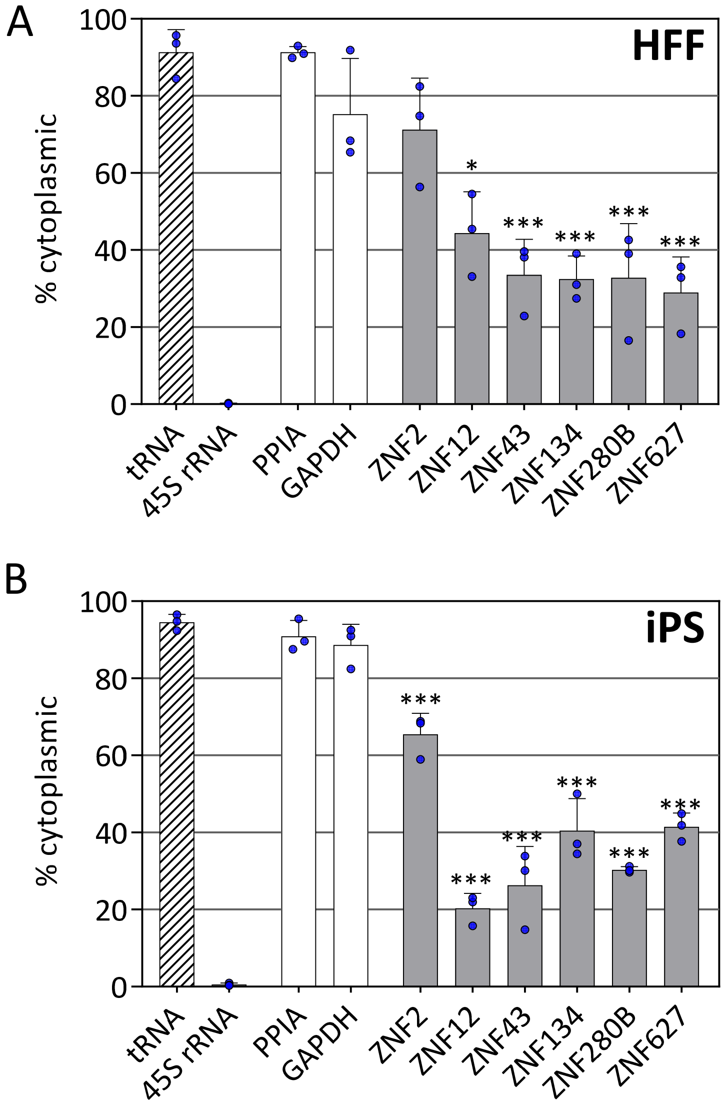

Supplementary Figure 4: C2H2-ZNF mRNAs are restricted to the nucleus in normal cell lines. RNA was isolated from the nucleus and cytoplasm of HFF (A) or iPS (B) cells and the abundance of the indicated transcripts in each fraction was determined by qRT-PCR. The percentage of each mRNA in the cytoplasmic fraction is shown. Error bars represent standard deviations. Blue circles represent individual replicates. Asterisks indicate significant differences from the PPIA and GAPDH controls (* $p<0.05$, *** $p<0.001$, one-way ANOVA, Tukey post-hoc test).

Supplementary Figure 5

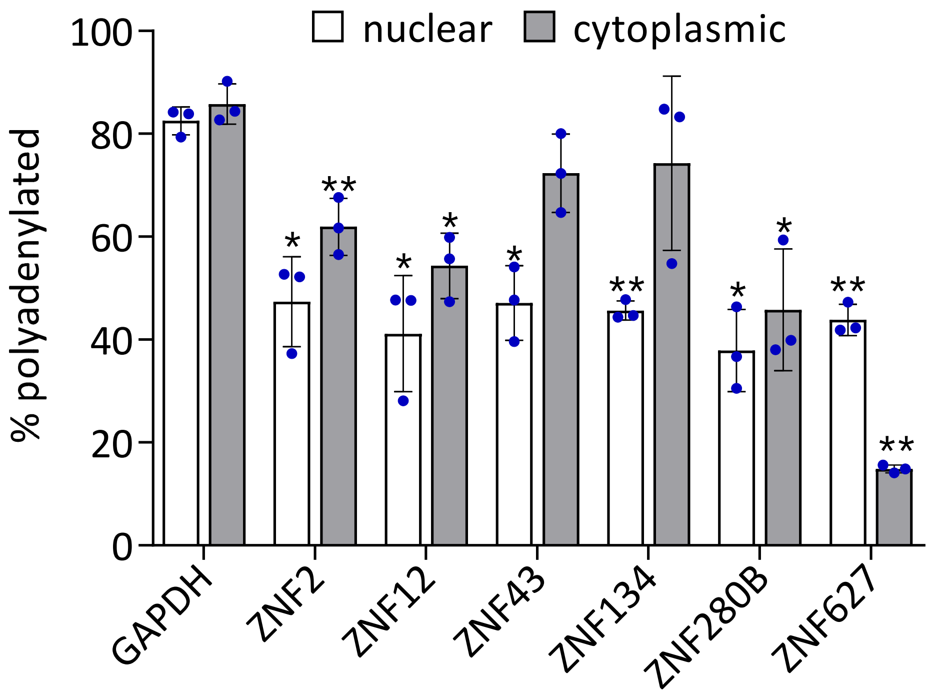

Supplementary Figure 5: Evaluation of polyadenylation levels in the nucleus and cytoplasm. HeLa cell nuclei and cytoplasm were separated and total RNA was isolated. The total RNA from each fraction was then further fractionated into poly(A)+ and poly(A)- fractions by binding to oligo(dT). The relative amount of the indicated transcripts in each fraction was assessed by qRT-PCR. For each group (nucleus and cytoplasm) a one way ANOVA followed by Dunnett's test for multiple comparisons was used to determine whether ZNF mRNAs behaved differently than GAPDH mRNA (which is predominantly polyadenylated in both fractions). Samples marked with asterisks showed significantly less polyadenylation than GAPDH mRNA in the same fraction.
* $p<0.05$, ** $p<0.005$.

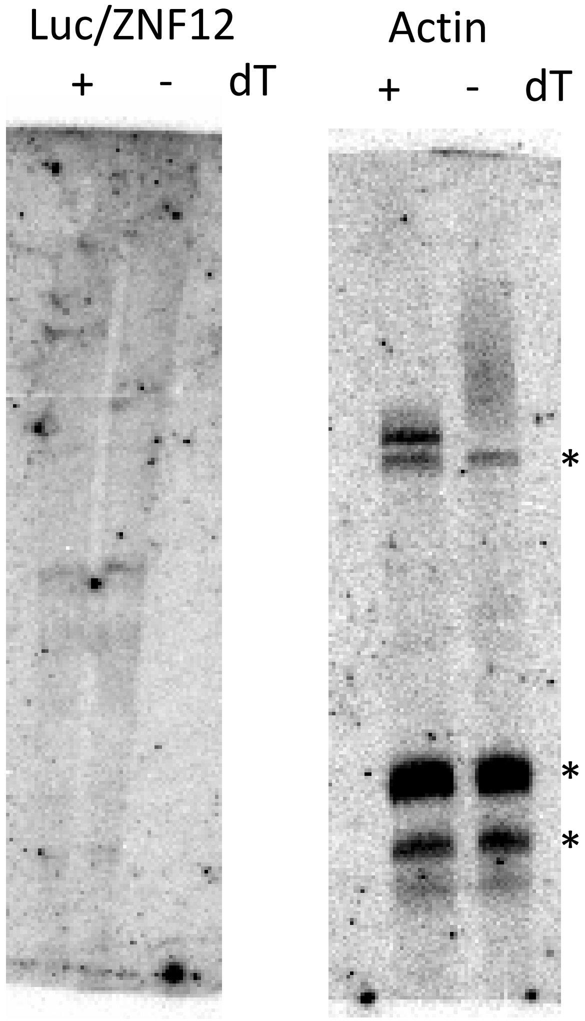

Supplementary Figure 6: Uncropped images of northerns shown in Figure 3D.
* indicates non-specific bands

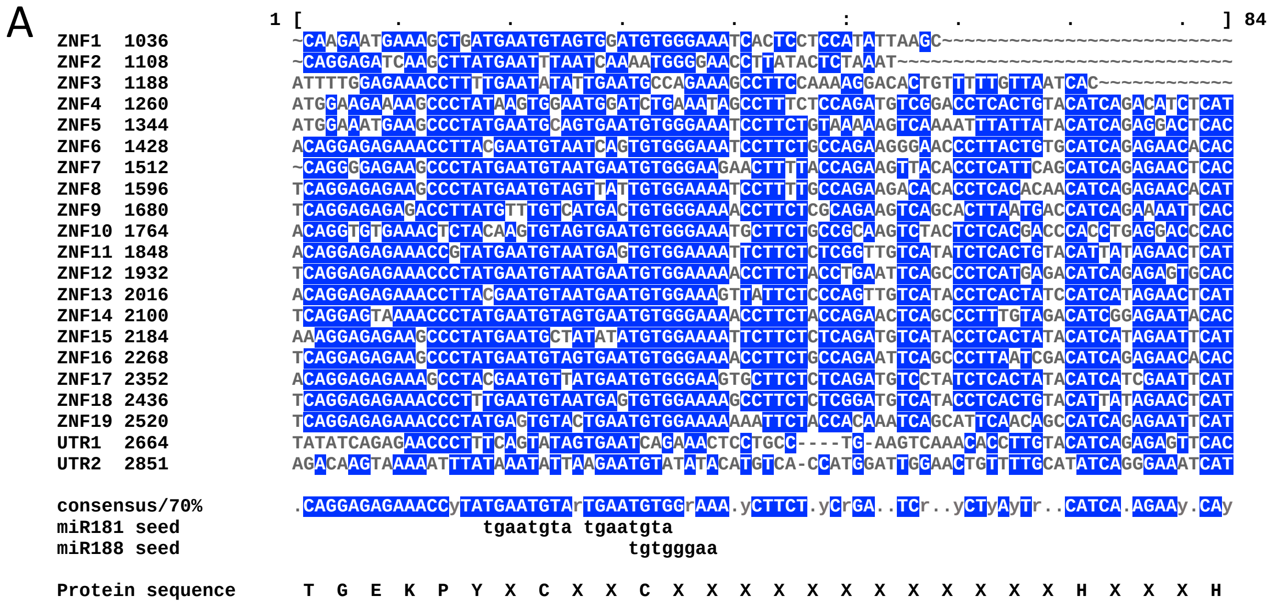

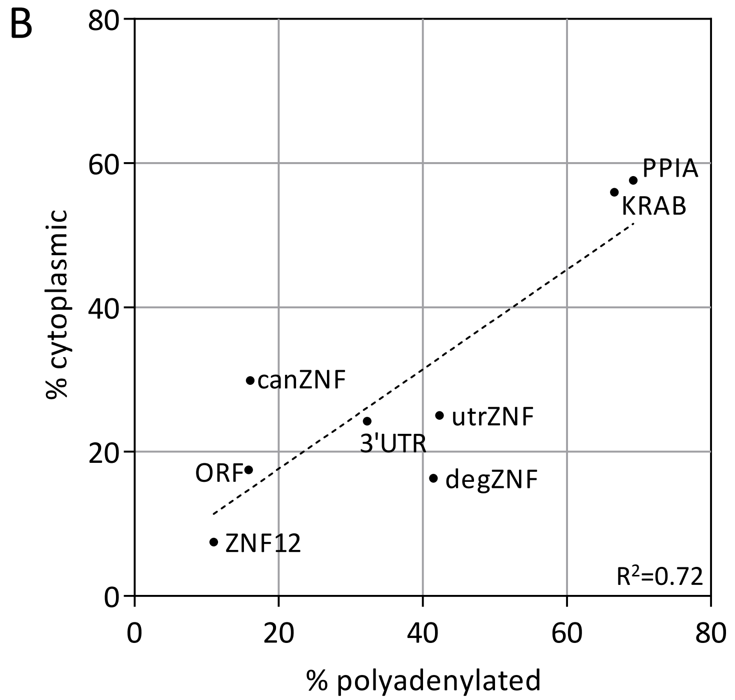

Supplementary Figure 7: A: Alignment of the 84 nt long repeated elements within the ORF and UTR of ZNF12. Nucleotides identical to the consensus for this set are highlighted in blue. The positions of miR-181 and miR188 seed matches are also shown along with the canonical protein sequence of the C2H2 ZNF motif. Alignment was accomplished with Clustal Omega and colored using Mview. B: For each reporter transcript, the percentage found in the cytoplasm was plotted against the percentage bound to oligo(dT)

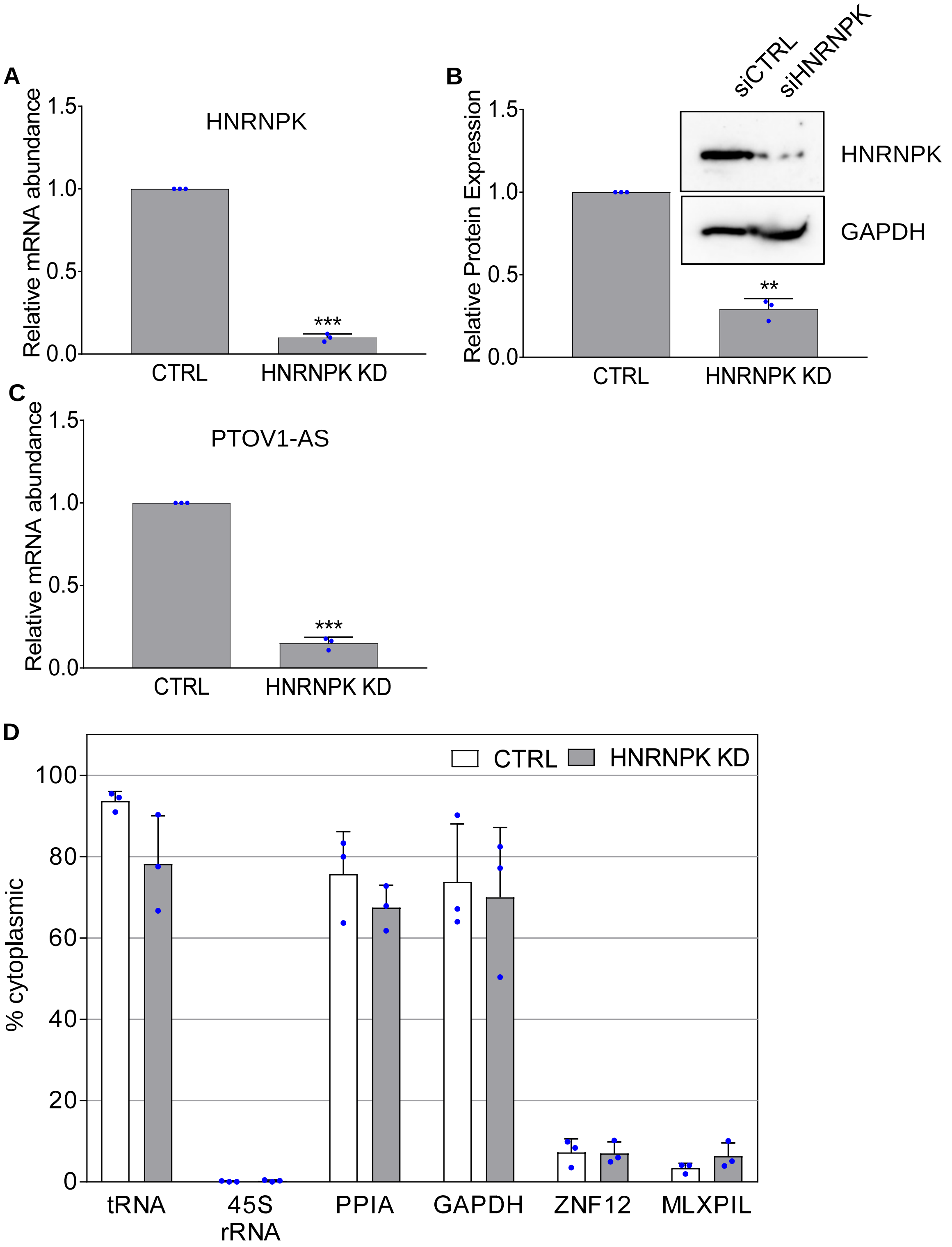

Supplementary Figure 8: HnRNP K depletion does not influence nuclear retention of ZNF mRNAs. HeLa cells were transfected with siRNAs targeting hnRNP K or a a control siRNA and abundance of the hnRNP K mRNA and protein was determined by qRT-PCR (A) and western blotting (B). The abundance of a known hnRNP K target, PTOV-AS1 was assessed by qRT-PCR (C). Forty eight hours after siRNA transfection, HeLa cells were fractionated to separate the nucleus and cytoplasm and the abundance of the indicated transcripts was determined in each fraction by qRT-PCR. Error bars represent standard deviations. ***p<0.0005, ** p<0.05 by t-test.

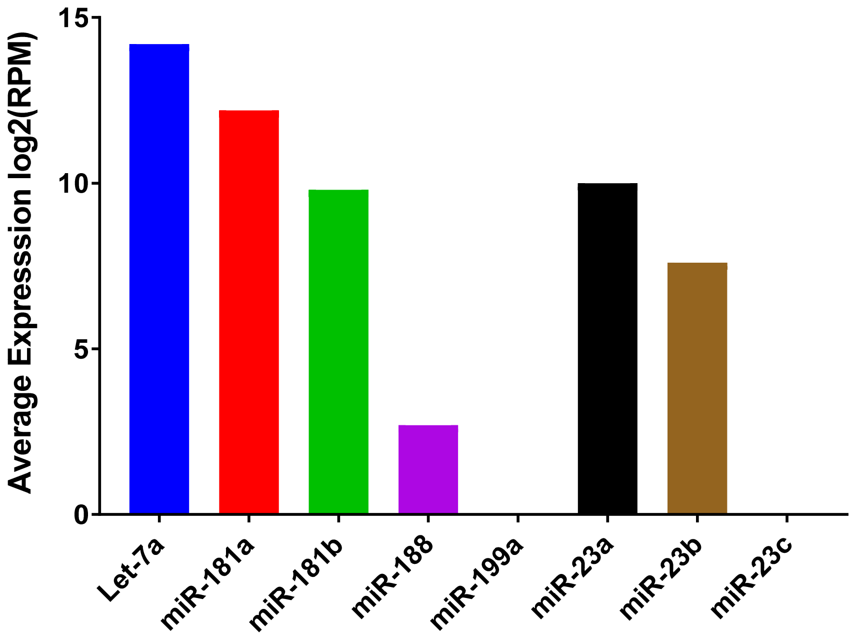

Supplementary Figure 9: miRNAs targeting ZNF mRNAs are expressed in HeLa cells. Expression data derived from HeLa cells for let-7a (an abundant miRNA), and for miRNAs targeting ZNF coding regions was retrieved from miRmine (Panwar et al, 2017).

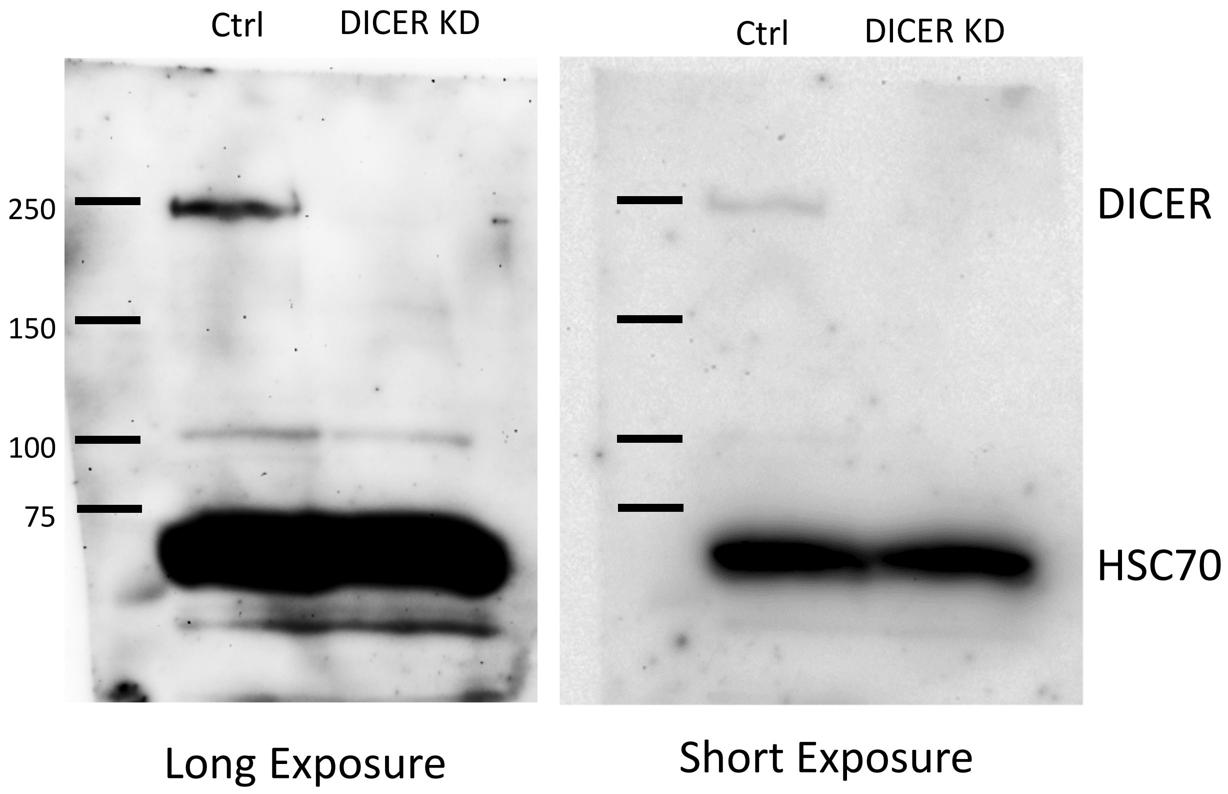

Supplementary Figure 10: Uncropped western blot showing knockdown of DICER (related to Figure 5)
